# Metagenomic Characterization of Gut Microbiota in Children with Autism Spectrum Disorder: Microbial Signatures and Modulation by Anti-Inflammatory Diet and Probiotics

**DOI:** 10.3390/ph18091376

**Published:** 2025-09-15

**Authors:** Marysol Valencia-Buitrago, Rodrigo Dias Oliveira-Carvalho, Valbert Cardoso, Jessica Triviño-Valencia, Luisa Matilde Salamanca-Duque, Vanessa Martínez-Díaz, Jovanny Zabaleta, Narmer Fernando Galeano-Vanegas, Carlos Andrés Naranjo-Galvis

**Affiliations:** 1 Facultad de Salud, Universidad Autónoma de Manizales, Manizales 170004, Colombia; 2 Faculty of Pharmacy, Universidade Federal de Minas Gerais, Belo Horizonte 31270-901, Brazil; 3 Laboratório de Radioisótopos, Departamento de Análises Clínicas e Toxicológicas, Faculdade de Farmácia, Campus Pampulha, Universidade Federal de Minas Gerais, Belo Horizonte 31270-901, Brazil; 4 Department of Interdisciplinary Oncology, Louisiana State University Health Sciences Center, New Orleans, LA 70112, USA; 5 Centro de Bioinformática y Biologia Computacional de Colombia (BIOS), Manizales 170004, Colombia

**Keywords:** autism spectrum disorder, gut microbiota, anti-inflammatory diet, probiotics, metagenomics, gastrointestinal disorders, Colombian children

## Abstract

**Background:** Autism Spectrum Disorder (ASD) is increasingly associated with alterations in gut microbiota, intestinal permeability, and immune dysregulation. However, integrative studies exploring these mechanisms in Latin American populations are lacking. **Objective:** To characterize gut microbiota profiles in Colombian children with ASD and evaluate the effects of two microbiota-targeted interventions, an anti-inflammatory diet and a probiotic formulation, on microbial diversity and taxonomic composition. **Methods:** In a two-phase study, shotgun metagenomic sequencing was performed on fecal samples from 23 children with ASD and 7 typically developing (TD) controls. In the second phase, 17 children with ASD were randomized to receive a 12-week intervention (anti-inflammatory diet, probiotics, or no intervention). Alpha diversity indices (Shannon, Pielou, and Chao1) and differential abundance analyses were conducted. **Results:** Compared to TD children, those with ASD showed a higher *Firmicutes/Bacteroidetes* ratio and a significantly increased abundance of genera such as *Clostridioides*, *Thomasclavelia*, *Alistipes*, and *Coprococcus*. The presence of functional gastrointestinal disorders (FGIDs) in ASD patients is associated with reduced microbial richness. POST-intervention, the anti-inflammatory diet group showed that no statistically significant changes in alpha diversity were observed, although a slight upward trend was noted and significant enrichment of six bacterial genera, including *Moraxella* and *Eubacterium*. The probiotic group exhibited a significant increase in *Romboutsia* and a decrease in *Lachnospira*. Cytokine–microbiota networks in ASD were fragmented and dominated by IFN-γ and MCP-1 hubs, indicating systemic immune activation. Interventions induced functional remodeling: The anti-inflammatory diet increased the number of beneficial genera (*Eubacterium*, *Adlercreutzia*) and shifted networks toward positive correlations involving IL-8 and MIP-1β. Probiotics increased *Romboutsia*, reduced *Lachnospira*, and restructured networks with regulatory cytokines (SDF-1α, Eotaxin) and SCFA-producing taxa (*Blautia*, *Roseburia*). **Conclusions:** Children with ASD in Colombia displayed distinct microbial profiles characterized by pro-inflammatory taxa and altered richness. Both the anti-inflammatory diet and probiotics produced compositional shifts in the gut microbiota, although global changes in diversity were limited. These findings support the potential of microbiota-targeted nutritional strategies for ASD and underscore the need for precision interventions tailored to specific clinical and microbial phenotypes.

## 1. Introduction

Autism Spectrum Disorder (ASD) is a neurodevelopmental condition characterized by difficulties in social communication and the presence of restricted, repetitive, and inflexible patterns of behavior, typically manifesting in early childhood [1,2]. Its prevalence has increased in recent decades, potentially due to changes in diagnostic criteria, earlier identification, and heightened awareness among healthcare professionals [3,4]. Nevertheless, the clinical complexity of ASD continues to hinder its accurate diagnosis, requiring approaches that account for the heterogeneity of symptoms and underlying neurobiological mechanisms [5]. According to the World Health Organization (WHO), one in every 100 children worldwide is affected by autism [6]. In Colombia, the Clinical Protocol for the Diagnosis, Treatment, and Comprehensive Care Pathway for Children with ASD (2015) reported that approximately 16% of the population under the age of 15 presented with some form of developmental disorder, including ASD, although no recent official national data are available [7]. García-Zambrano et al. [8] estimated a prevalence of 18.7 per 10,000 children receiving healthcare services in 2019, representing a substantial increase compared to 2009 (4.2/10,000), particularly in the Andean and Caribbean regions. Moreover, a higher prevalence has been consistently documented in males, with a male-to-female ratio of 8:1; however, when females are diagnosed, they tend to exhibit a more severe phenotype, often associated with greater intellectual impairment [9,10].

Although the etiology of ASD remains unclear, it is estimated that 40–80% of cases are attributable to genetic factors, while the remaining risk is linked to environmental influences, primarily exerted through epigenetic regulatory mechanisms [11]. Among these, maternal immune activation (MIA), which is triggered by infections or autoimmune conditions during pregnancy, has received particular attention. MIA is associated with oxidative stress and mitochondrial dysfunction, which may disrupt critical neurodevelopmental processes such as neurogenesis, gliogenesis, synaptogenesis, and blood–brain barrier formation [12] However, beyond intrauterine development, a dysregulated immune response characterized by excessive inflammation has been proposed to persist throughout the lifespan of individuals with ASD [13,14,15,16], partially mediated by increased expression and activation of toll-like receptor 4 (TLR-4) and the nuclear transcription factor NF-κB [17,18,19]. However, the mechanism underlying these changes remains unclear. Environmental agents, including viruses and bacteria, may act upon pre-existing immunogenetic vulnerabilities conferred by inherited genetic variations [20].

A potential mechanism implicated in TLR-4 receptor activation in individuals with ASD is exposure to lipopolysaccharide (LPS), an endotoxin present in the outer membrane of gram-negative bacteria that forms part of the gut microbiota, with elevated levels reported in this population [21]. Increased LPS concentrations, along with specific colony-forming units (CFUs), contribute to the overactivation of the TLR-4 signaling pathway [19], which has been associated with structural and functional alterations in the intestine, such as increased intestinal permeability [22], a condition also documented in individuals with autism [23]. In this context, upregulated expression of pore-forming proteins, such as *CLDN-1*, *OCLN*, and *TRIC*, and downregulation of barrier-forming proteins, such as *CLDN-2*, *CLDN-10*, and *CLDN-15*, have been observed [24]. This dysfunction facilitates LPS translocation, which, through interactions with immune cells (monocytes, macrophages, and polymorphonuclear leukocytes), activates the TLR4/MyD88/NF-κB signaling cascade, promoting a proinflammatory microenvironment associated with ASD [25,26]. These findings support the hypothesis that individuals with autism exhibit impaired intestinal barrier integrity and dysbiosis, which together disrupt the gut–brain axis; however, it remains unclear whether dysbiosis is a cause or consequence of ASD [27]. This is a critical question, given the bidirectional interaction between microbiota and mucosal immunity, whereby immune responses shape microbial composition through mechanisms such as the secretion of antimicrobial molecules, including REGIIIa, α-defensins, β-defensins, IgA, and commensal-specific T cells, as well as inflammatory pathways that selectively promote the growth of specific bacterial species [28].

Several studies have reported alterations in the gut microbiota of individuals with ASD, including reduced bacterial diversity and an altered *Firmicutes*/*Bacteroidetes* ratio [27]. Increases in *Actinobacteria* and *Proteobacteria* have also been documented [29], along with the presence of bacteria such as *Clostridia*, *Desulfovibrio*, *Sutterella*, and *Ruminococcus*, which produce propionic acid (PPA). This short-chain fatty acid has been linked to the induction of proinflammatory cytokines via the NF-κB pathway, reduction in endogenous antioxidant levels, and mitochondrial dysfunction [30]. Further studies have demonstrated associations between microbial profiles and immune pathways [31], including correlations between *Clostridium* species and proinflammatory cytokines [32]. Additionally, children with ASD and gastrointestinal symptoms have shown a decrease in butyrate-producing bacteria (*Faecalibacterium* and *Coprococcus*) and an increase in potentially pathogenic species (*Enterococcus* and *Eggerthella*) [33]. These findings suggest the existence of a distinct microbial profile in ASD that is associated with altered neuroimmune signaling and chronic inflammatory processes. Although the current evidence remains preliminary, the potential of omics-based approaches to advance precision medicine is being increasingly recognized [34].

Within this framework, diet has emerged as a key environmental factor influencing the development of altered microbial profiles and reduced bacterial diversity in children with ASD [35]. These children often exhibit restrictive eating behaviors, food selectivity, dietary monotony, and aversions to foods based on sensory characteristics, resulting in nutrient-deficient diets lacking proteins, calcium, vitamin D, thiamine, riboflavin, vitamin B12, among others [36]. Such inadequate nutritional intake may exacerbate ASD pathogenesis [37], given that food provides substrates that regulate and modify the gut microbiota composition, which in turn modulates peripheral and central inflammation [38,39]. These changes can affect the gut–brain axis and contribute to the manifestation of signs and symptoms through diverse physiological signaling pathways [40,41]. In other words, low-grade chronic inflammation observed in children with ASD may be linked to proinflammatory dietary patterns stemming from selective eating behaviors and hypersensitivity to specific foods [42].

Consequently, dietary interventions and nutritional supplements have received increasing attention in research on ASD. For instance, Mohammad et al. [43] developed a computational model demonstrating that a high-fiber diet, in combination with probiotics such as *Lactobacillus acidophilus*, *Bifidobacterium longum*, *Akkermansia muciniphila*, and *Prevotella ruminicola*, reduces oxidative stress in both the gut and brain. A meta-analysis by Siafis et al. [44] reported the potential benefits of supplements, such as carnosine, omega-3 fatty acids, sulforaphane, and probiotics, although the findings were inconclusive. Li et al. [45] highlighted the improvement of gastrointestinal symptoms linked to *Clostridium* following treatment with antibiotics or probiotics such as *Bacteroides fragilis*. He et al. [46] suggested that probiotic mixtures, particularly those comprising the *Lactobacillus* genus, might improve behavioral symptoms through the production of gamma-aminobutyric acid (GABA). Gul et al. [47] described multiple mechanisms of probiotic action, including anti-inflammatory effects and modulation of the gut–brain axis, but emphasized the need for additional clinical evidence. Similarly, Bakırhan et al. [48] reported that a Mediterranean diet may improve gastrointestinal health and quality of life in children with disabilities including ASD. A systematic review published in 2022 examined antioxidant- and polyphenol-rich diets as potential ASD treatments, highlighting that these compounds are metabolized by the gut microbiota into bioactive antioxidant metabolites. However, while such diets may alleviate autism symptoms, the current evidence is insufficient to support their exclusive use as therapeutic strategies [49]. These findings contribute to the expanding body of research on the interplay between diet, the microbiota, and ASD.

In light of this evidence, dietary interventions are regarded as promising therapeutic strategies for managing inflammation-related health conditions. However, further research is needed to determine the most appropriate dietary approach based on underlying biological interactions. Notably, to date, no studies have explored such interventions in the Colombian context, a relevant gap given the influence of geographical and cultural factors on microbial composition [31]. Investigating the effects of dietary interventions in ASD should not be limited to behavioral outcomes [44,46,48,49,50,51,52] but should also aim to elucidate the underlying biological mechanisms. Most available studies focus on clinical variables, such as behavior, communication, repetitive behaviors, gastrointestinal symptoms, or sleep, without providing detailed insight into the biological changes that support the plausibility and efficacy of these interventions. In Colombia, research on ASD from a basic scientific perspective remains limited, and no studies have identified the use of omics-based approaches or next-generation sequencing to characterize biological markers or identify therapeutic targets. Considering Colombia’s geographic and agricultural diversity, it is crucial to establish a foundational understanding of how diet influences the local population and extend this inquiry to other neurodevelopmental disorders associated with low-grade chronic inflammation.

Moreover, given the intricate bidirectional communication between the immune system and gut microbiota, integration of immunological markers with metagenomic data is crucial for understanding the pathophysiology of ASD. In particular, cytokines serve as essential mediators of neuroimmune interactions and are strongly influenced by microbial composition and activity. By analyzing the relative abundance of gut bacterial genera and their correlations with circulating cytokine levels, this study adopts a perspective to explore how dietary and probiotic interventions may reshape host–microbiota–immune networks in children with ASD. These integrated analyses aim to move beyond descriptive taxonomic profiles in order to elucidate the potential immunomodulatory pathways affected by microbial shifts. Therefore, it is necessary to investigate the biological effects of an anti-inflammatory diet composed of regionally available foods to evaluate its influence on gut microbiota composition [53,54].

Such research could facilitate the development of personalized nutritional medicine strategies tailored to Colombia. Accordingly, this study was conducted in two phases: the first aimed to characterize and compare the gut microbiota composition of children diagnosed with ASD and typically developing (TD) children through metagenomic profiling using shotgun sequencing; the second aimed to evaluate the impact of two microbiota-targeted interventions, namely, a probiotic formulation and an anti-inflammatory diet, on gut microbiota composition in children with ASD, based on PRE- and POST-intervention metagenomic analyses.

## 2. Results

### 2.1. Sociodemographic Characteristics of Study Participants

A total of 23 children with ASD and 7 TD children were included in the study. The ASD group had a higher proportion of males (86.9%) than the TD group (57.1%), and the mean age was slightly lower in the ASD group (10.3 years) than in the TD group (12.1 years). Functional gastrointestinal disorders (FGIDs) were reported in 65.2% and 57.1% of patients in the ASD and TD groups, respectively. In terms of height-for-age, 73.9% of the children with ASD had adequate growth, while 26.1% showed either a risk of delay or low height. The BMI-for-age classifications showed greater variability in the ASD group, with 39.1% having an adequate weight status and the remainder presenting risk of thinness (21.7%), thinness (4.3%), overweight (21.7%), or obesity (13.0%). In contrast, the TD group showed no cases of thinness, with 57.1% of participants having an adequate BMI, 14.3% classified as overweight, and 28.6% as obese. The sociodemographic and nutritional characteristics of the patients are summarized in Table 1.

The second phase of the study, corresponding to the experimental design involving microbiota-targeted interventions, was completed by 17 participants with ASD. The participants were divided into three parallel groups: control (n = 6), anti-inflammatory diet (n = 5), and probiotics (n = 6). Table 2 presents the sociodemographic characteristics of each group, including sex, age, presence of functional gastrointestinal disorders (FGIDs), and nutritional status based on height-for-age and body mass index (BMI)-for-age classifications. Although all groups were similar in average age, notable differences were observed in the sex distribution and FGID prevalence. The probiotic group consisted exclusively of male participants and reported the highest frequency of FGIDs (100%), followed by the control group (83.3%), whereas the diet group had the lowest prevalence (40%). In terms of height-for-age, all children in the diet group showed adequate growth compared to 50.0% in the control group and 83.3% in the probiotics group. BMI-for-age classifications varied among the groups, with the diet group showing a higher proportion of participants classified as thin or overweight.

### 2.2. Comparative Analysis Between Children with ASD and TD

A general characterization of the gut microbiota was provided for both groups based on the relative abundance of bacterial taxa at the phylum and genus levels. In addition, intergroup comparisons were conducted to evaluate alpha diversity indices such as richness, evenness, and overall diversity, as well as the relative abundance of bacterial species. Special emphasis was placed on taxa that exhibited statistically significant differences between the ASD and TD groups, highlighting potential microbial signatures associated with ASD.

#### 2.2.1. Taxonomic Composition at the Phylum and Genus Levels

**Figure 1:**
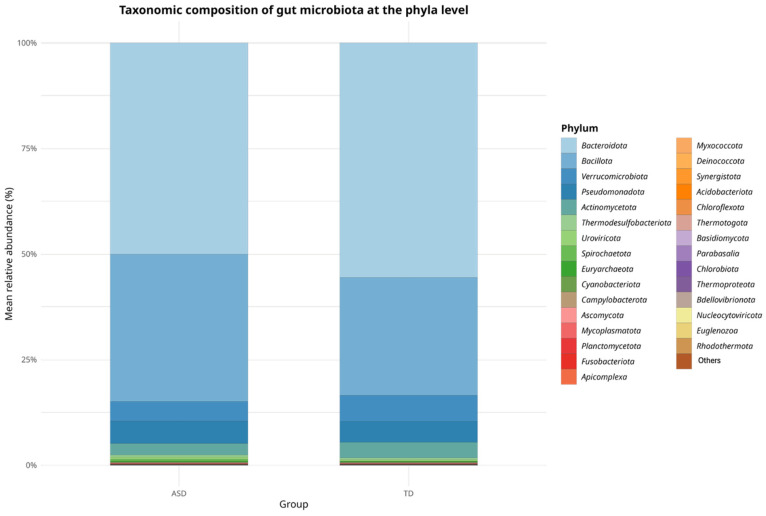
Taxonomic composition of gut microbiota at the phylum level in children with Autism Spectrum Disorder (ASD) and typically developing (TD) controls. Stacked bar plots represent the mean relative abundance (%) of bacterial phyla across groups. *Bacteroidota* and *Bacillota* were the predominant phyla in both groups, with a higher *Bacteroidota*/*Bacillota* ratio observed in ASD children, suggesting a microbial imbalance potentially associated with gut dysbiosis.

In terms of relative abundance at the phylum level, both the ASD and TD groups shared a broadly similar microbial composition, as shown in Figure 1. *Bacteroidetes* and *Bacillota* (formerly *Firmicutes*) were the dominant phyla in both groups, representing the majority of the gut microbial community. *Bacteroidetes* appeared to be more predominant in the TD group, whereas *Bacillota* showed a higher proportion in the ASD group. Quantitatively, the *Bacillota-to-Bacteroidota* (*Firmicutes*/*Bacteroidetes*, F/B) ratio was higher in the ASD group (0.71) than in the TD group (0.48), suggesting a relative increase in this balance among children with ASD, which may reflect alterations in the gut microbial ecology frequently associated with dysbiosis.

**Figure 2:**
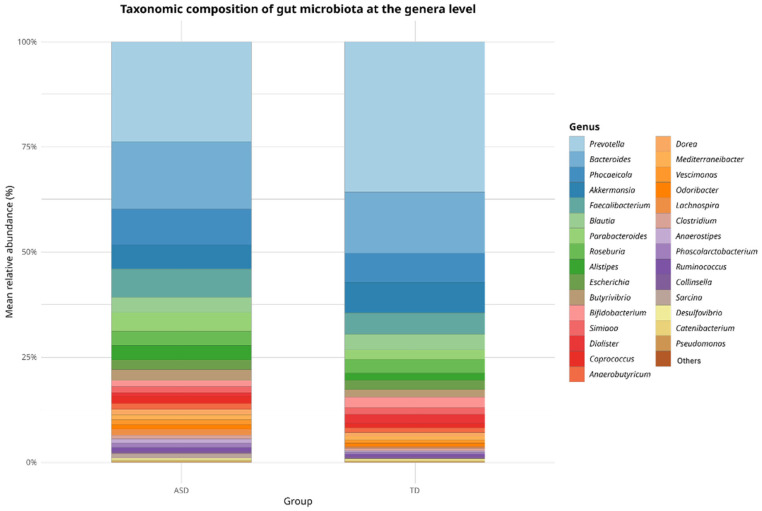
Taxonomic composition of gut microbiota at the genus level in children with Autism Spectrum Disorder (ASD) and typically developing (TD) controls. Stacked bar plots show the mean relative abundance (%) of the most prevalent bacterial genera. Genera such as *Prevotella*, *Bacteroides*, *Phocaeicola*, and *Faecalibacterium* dominated both groups, with a higher representation of *Prevotella* and *Bacteroides* and a lower abundance of butyrate-producing genera (*Roseburia*, *Blautia*, *Butyricicoccus*) in ASD children, suggesting altered microbial functions related to gut homeostasis and short-chain fatty acid metabolism.

In terms of relative abundance at the genus level, both groups (ASD and TD) exhibited a broadly similar microbial composition dominated by a shared set of core genera, as shown in Figure 2. Genera such as *Prevotella*, *Bacteroides*, *Phocaeicola*, *Akkermansia*, *Faecalibacterium*, *Blautia*, *Parabacteroides*, *Roseburia*, and *Alistipes* were among the most prevalent in both the groups. However, slight variations in the relative abundance of the less dominant genera were observed, which may indicate compositional shifts potentially associated with ASD. Although these differences were not statistically significant at this taxonomic level, they provide a descriptive overview that supports subsequent analyses focused on identifying discriminant taxa.

#### 2.2.2. Alpha Diversity, Evenness, and Richness Indices

A comparative analysis of alpha diversity, evenness, and richness indices between children with ASD and TD children is presented in Table 3. Normality assumptions were satisfied for the TD group across all indices, whereas the ASD group showed a non-normal distribution of the Chao1 richness index. Levene’s test confirmed the homogeneity of variances for both the Shannon and Pielou indices, which justified the use of *t*-tests for these comparisons. No statistically significant differences were observed in Shannon diversity (*p* = 0.2129) or Pielou’s evenness (*p* = 0.2118). Given the violation of normality, the Chao1 index was analyzed using the Mann–Whitney U test, which also showed no significant difference between groups (*p* = 0.2023). Overall, these findings indicate that the global structure of the gut microbiota, in terms of diversity, evenness, and richness, did not significantly differ between children with ASD and their neurotypical peers (Table 3).

**Figure 3:**
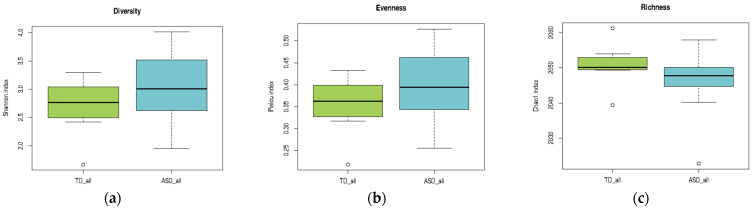
Alpha diversity comparison between children with ASD and those with TD. (**a**) Shannon index (diversity), (**b**) Pielou index (evenness), and (**c**) Chao1 index (richness). Boxplots represent the distribution of the values for each group. Although no significant differences were detected, visual trends were observed.

As illustrated in Figure 3, the ASD group exhibited a broader range of values across all three indices than the TD group. In panel (a), the Shannon diversity index tended to be higher in the ASD group, suggesting greater microbial diversity, although the variability was also more pronounced. In panel (b), Pielou’s evenness index showed a slight increase in the ASD group, indicating a more evenly distributed microbial community. In contrast, panel (c) reveals that richness, as measured by the Chao1 index, was slightly lower in the ASD group than in the TD group. Although these trends did not reach statistical significance, they highlight potential differences in the microbial community structure that merit further investigation in larger cohorts.

#### 2.2.3. Differential Abundance of Bacterial Genera Between ASD and TD Groups

This section presents the bacterial genera that exhibited statistically significant differences in relative abundance between ASD and TD groups. The analysis was conducted using the STAMP software (v2.1.3), which allowed for pairwise comparisons with appropriate statistical corrections for multiple testing. Only genera with significant *p*-values were reported. The results were visualized through heatmaps and extended error bar plots to highlight group-level differences in abundance and effect sizes.

**Figure 4:**
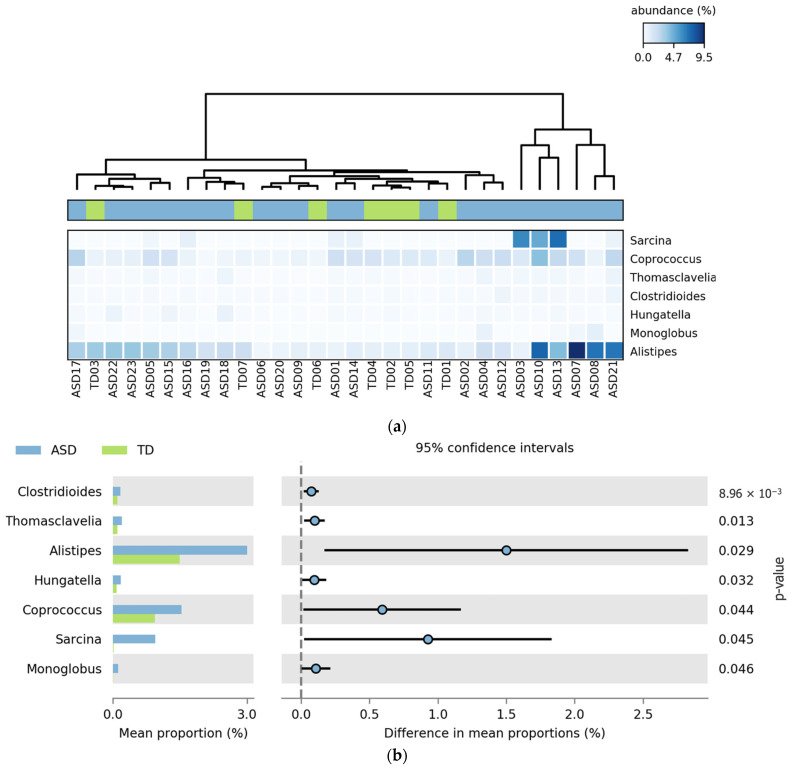
Differential abundance of bacterial genera in ASD and TD groups. (**a**) Heatmap showing the relative abundance (%) of bacterial genera that were significantly different between ASD without FGIDs and TD without FGIDs. Columns represent individual participants, and rows indicate bacterial genera. The color scale reflects relative abundance: lighter shades of blue correspond to lower abundance, while darker shades correspond to higher abundance levels. Normalization was applied across samples to enhance comparability. (**b**) Extended error bar plot comparing the mean proportions of significantly different genera between groups with 95% confidence intervals.

Figure 4 shows the bacterial genera that showed statistically significant differences in relative abundance between the ASD and TD groups. All seven genera, *Clostridioides*, *Thomasclavelia*, *Alistipes*, *Hungatella*, *Coprococcus*, *Sarcina*, and *Monoglobus*, were more abundant in the ASD group. The heatmap (Figure 4a) reveals clustering patterns with increased signal intensity in ASD participants, indicating consistent enrichment of these taxa across individuals. In the extended error bar plot (Figure 4b), the largest differences in the mean proportions were observed for *Alistipes*, *Coprococcus*, and *Sarcina*, all with *p*-values below 0.05.

### 2.3. Comparative Analysis Between Children with ASD and TD Stratified by the Presence or Absence of FGIDs

To explore whether the presence of functional gastrointestinal disorders (FGIDs) modulates gut microbiota composition within and across neurodevelopmental categories, participants were stratified into four subgroups based on diagnosis and FGID status: children with autism spectrum disorder without FGIDs (ASD, n = 8), children with ASD and FGIDs (ASD_FGIDs, n = 15), typically developing children without FGIDs (TD, n = 3), and TD children with FGIDs (TD_FGIDs, n = 4). Comparative analyses were conducted to evaluate differences in microbial diversity, evenness, richness, and taxonomic composition across these subgroups. The goal of this study was to assess whether gastrointestinal symptomatology exerts distinct effects on the gut microbiota depending on the neurodevelopmental context.

#### 2.3.1. Alpha Diversity, Evenness, and Richness Indices

The analysis focused on alpha diversity metrics including Shannon (diversity), Pielou (evenness), and Chao1 (richness) indices. Normality and homogeneity of variances were assessed using the Shapiro–Wilk and Levene’s tests, respectively, to determine the appropriate statistical test for each comparison. Depending on these assumptions, either independent-sample *t*-tests or Mann–Whitney U tests were applied. As shown in Table 4, most of the comparisons did not yield statistically significant differences. However, a significant reduction in richness (Chao1 index) was observed in the ASD_FGIDs group compared to that in the TD group (*p* = 0.033), suggesting that the presence of FGIDs in children with ASD may be associated with a loss of microbial richness.

**Figure 5:**
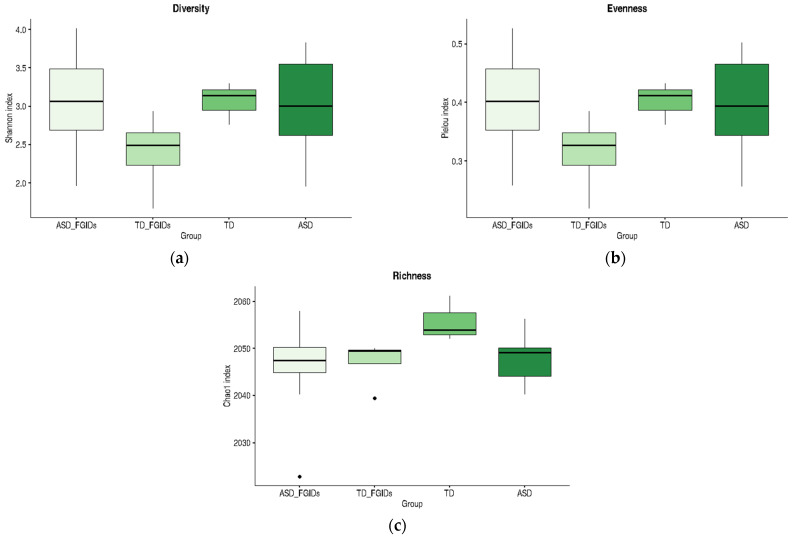
Comparison of alpha diversity metrics across ASD and TD subgroups stratified by the presence or absence of FGIDs. (**a**) Shannon index (diversity), (**b**) Pielou index (evenness), and (**c**) Chao1 index (richness). The boxplots represent the distribution of values for each group: ASD_FGIDs, TD_FGIDs, TD, and ASD.

Although the only statistically significant difference was observed between the TD and ASD_FGIDs groups for the Chao1 richness index, the boxplots in Figure 5 reveal consistent trends across all alpha diversity metrics. Notably, TD children without FGIDs showed the highest mean values for diversity, evenness, and richness, suggesting a more balanced and stable microbial community in the absence of gastrointestinal symptoms. In contrast, the presence of FGIDs in TD children was associated with marked reductions in Shannon and Pielou indices, indicating that gastrointestinal disturbances may negatively impact microbiota structure, even in neurotypical populations. However, among children with ASD, the presence or absence of FGIDs did not substantially alter the alpha diversity profiles; the mean values for diversity, evenness, and richness remained similar across all ASD subgroups. These patterns suggest that while FGIDs may be a key modulator of microbiota diversity in TD children, their influence on ASD appears less pronounced, possibly due to an already altered microbial baseline associated with the neurodevelopmental condition.

#### 2.3.2. Differential Abundance of Bacterial Genera Between ASD and TD Subgroups Stratified by FGID Status

##### Differential Abundance of Bacterial Genera: TD vs. ASD Without FGIDs

**Figure 6:**
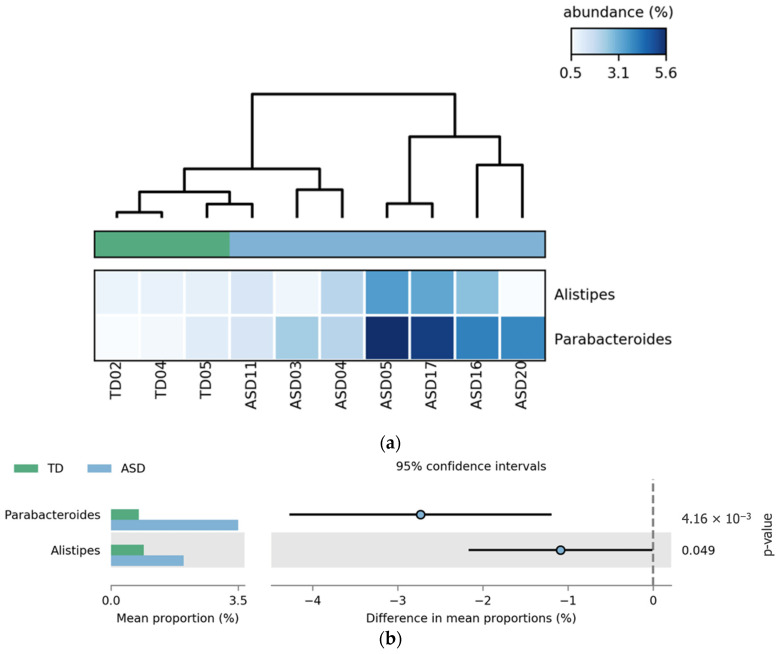
Differential abundance of bacterial genera in children with ASD and TD without FGIDs. (**a**) Heatmap showing the relative abundance (%) of bacterial genera that were significantly different between ASD without FGIDs and TD without FGIDs. Columns represent individual participants, and rows indicate bacterial genera. The color scale reflects relative abundance: lighter shades of blue correspond to lower abundance, while darker shades correspond to higher abundance levels. Normalization was applied across samples to enhance comparability. (**b**) Extended error bar plot comparing mean proportions of significant genera between the ASD and TD groups, showing 95% confidence intervals.

Comparative analysis revealed that the genera *Parabacteroides* and *Alistipes* were significantly more abundant in the ASD group without FGIDs than in the TD group without FGIDs. *Parabacteroides* showed the most pronounced difference (*p* = 4.16 × 10^−8^), followed by *Alistipes* (*p*-value = 0.049). These differences are illustrated in Figure 6, where the heatmap (a) highlights the consistent enrichment of both genera in ASD samples, and the extended error bar plot (b) confirms the statistical significance and direction of the effect.

##### Differential Abundance of Bacterial Genera: TD vs. TD_FGIDs

**Figure 7:**
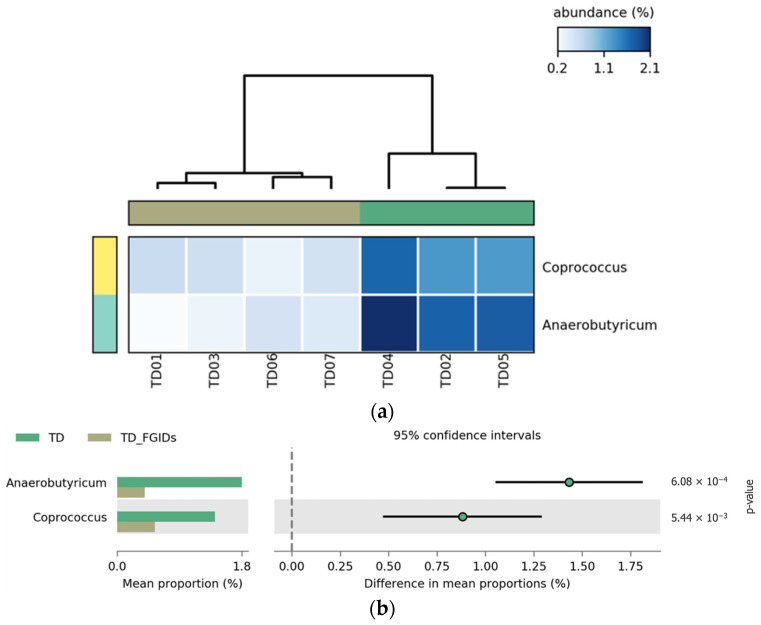
Differential abundance of bacterial genera between typically developing (TD) children with and without functional gastrointestinal disorders (FGIDs). (**a**) Heatmap representing the relative abundance (%) of bacterial genera significantly different between TD children with and without FGIDs. Each column corresponds to one participant, and each row corresponds to a bacterial genus. The color gradient illustrates relative abundance, with lighter shades of blue denoting lower abundance and darker shades indicating higher abundance values. Relative abundance is normalized across groups for comparability. (**b**) Extended error bar plot showing differences in the mean proportions of the identified genera, including 95% confidence intervals.

A comparison between TD children with and without FGIDs revealed significant differences in the relative abundance of two bacterial genera, *Anaerobutyricum* and *Coprococcus*. Both genera were more abundant in TD children without FGIDs, suggesting a possible protective or health-associated microbial profile. These genera are known butyrate producers, and have been associated with gut barrier integrity and anti-inflammatory effects. The presence of FGIDs in TD children is associated with a marked depletion of these beneficial taxa, which may reflect gut microbial dysbiosis linked to gastrointestinal symptomatology. These results, shown in Figure 7, show consistent patterns in the heatmap (a), and statistically significant group-level differences confirmed in the extended error bar plot (b), with *p*-values of 6.08 × 10^−4^ and 5.44 × 10^−3^, respectively.

##### Differential Abundance of Bacterial Genera: TD vs. ASD_FGIDs

**Figure 8:**
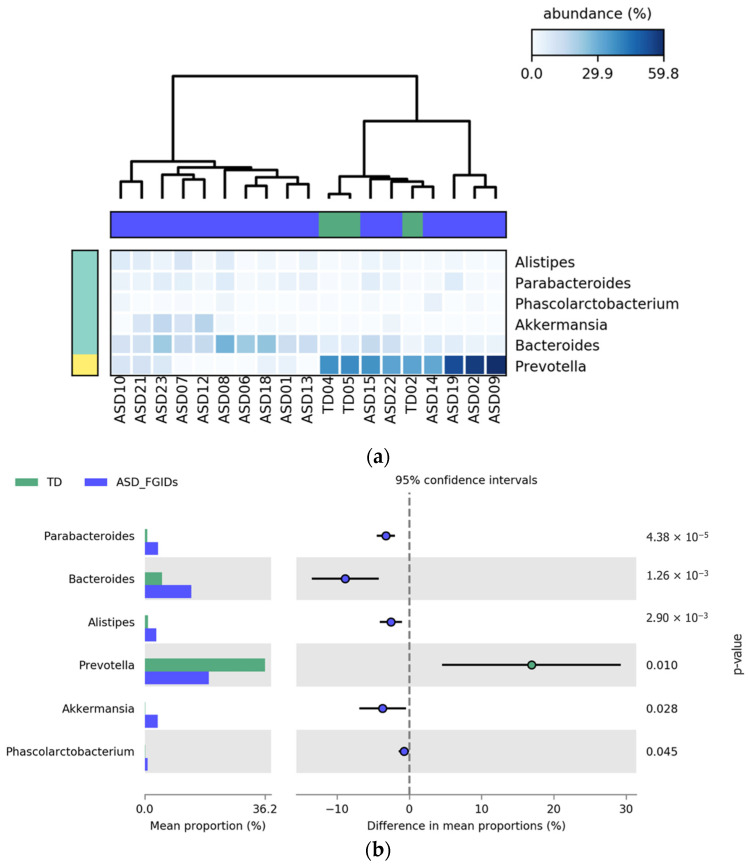
Differential abundance of bacterial genera between TD and ASD_FGIDs. (**a**) Heatmap displaying that the relative abundance (%) of bacterial genera was significantly different between children with ASD and FGIDs and TD children. Each column represents a participant, and each row represents a genus. The color scale indicates relative abundance, where lighter blue denotes lower levels and darker blue indicates higher abundance. Values are normalized across samples for consistency. (**b**) Extended error bar plot showing differences in mean proportions between groups, including 95% confidence intervals.

The comparison between TD children and those with ASD and FGIDs revealed significant differences in the relative abundances of the six bacterial genera. Among them, *Prevotella* was the only genus found in greater abundance in the TD group, potentially reflecting a more favorable or balanced microbial profile. In contrast, *Parabacteroides*, *Bacteroides*, *Alistipes*, *Akkermansia*, and *Phascolarctobacterium* were more abundant in the children with ASD_FGIDs. While *Prevotella* had the highest mean proportion overall, *Parabacteroides* showed the most statistically significant difference between the groups (*p* = 4.38 × 10^−5^), underscoring its potential role as a microbial biomarker in ASD with gastrointestinal comorbidities. As depicted in Figure 8, the heatmap (a) illustrates distinct clustering and relative abundance patterns, and the extended error bar plot (b) confirms the direction and significance of group-level differences.

##### Differential Abundance of Bacterial Genera: TD_FGIDs vs. ASD_FGIDs

**Figure 9:**
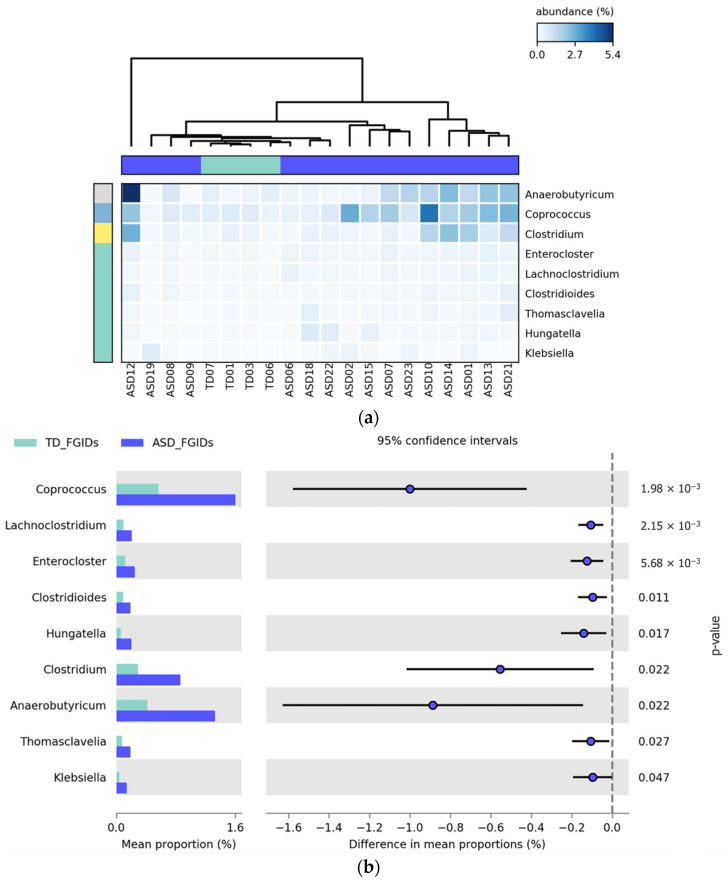
Differential abundance of bacterial genera between typically developing children with functional gastrointestinal disorders (TD_FGIDs) and children with autism spectrum disorder (ASD_FGIDs). (**a**) Heatmap illustrating the relative abundance (%) of bacterial genera significantly different between ASD with FGIDs and TD with FGIDs. Columns correspond to participants and rows to bacterial genera. The color scale indicates relative abundance, with lighter shades of blue corresponding to lower abundance and darker shades representing higher abundance. Normalization was applied across samples for visual clarity. (**b**) Extended error bar plot displaying differences in mean proportions for each genus with 95% confidence intervals and FDR-corrected *p*-values.

The comparison between children with ASD and FGIDs (ASD_FGIDs) and typically developing children with FGIDs (TD_FGIDs) revealed significant differences in the relative abundance of nine bacterial genera. All genera identified—*Coprococcus*, *Lachnoclostridium*, *Enterocloster*, *Clostridioides*, *Hungatella*, *Clostridium*, *Anaerobutyricum*, *Thomasclavelia*, and *Klebsiella*—were more abundant in the ASD_FGIDs group, suggesting a distinct microbial signature associated with the coexistence of ASD and gastrointestinal disturbances. Among these, *Coprococcus*, *Anaerobutyricum*, and *Clostridium* exhibited the greatest differences in mean proportions, potentially indicating a strong microbial imbalance in this subgroup. As illustrated in Figure 9, the heatmap (a) highlights the elevated abundance of these genera across multiple ASD_FGIDs samples, whereas the extended error bar plot (b) confirms the statistical significance and direction of the observed differences, with all genera reaching FDR-corrected *p*-values below 0.05.

### 2.4. Comparative Analysis of the Gut Microbiota in Children with ASD Before and After Dietary and Probiotic Interventions

To evaluate the effects of microbiota-targeted interventions on gut microbial diversity and composition, pre–post comparisons were conducted within the ASD group following two parallel strategies: an anti-inflammatory diet and a probiotic formulation. Seventeen children with ASD completed the 12-week intervention phase, distributed across three arms: ASD_control (n = 6), ASD diet (n = 5), and ASD_probiotics (n = 6). For each intervention arm, changes in alpha diversity indices (Shannon, Pielou, and Chao1) and bacterial taxonomic composition were analyzed based on paired fecal samples collected before and after the intervention. The aim of this study was to determine whether either strategy could modulate the gut microbiota in a directionally meaningful way compared to the baseline.

#### 2.4.1. Alpha Diversity, Evenness, and Richness Indices

The within-group comparison of the alpha diversity metrics before and after the 12-week intervention period is presented in Table 5. Across all three ASD subgroups (control, anti-inflammatory diet, and probiotics), no statistically significant changes were observed in the Shannon diversity or Pielou evenness indices. However, a significant difference was found in the Chao1 richness index within the ASD_control group (*p* < 0.05), indicating a change in microbial richness over time in the absence of active intervention. The Shapiro–Wilk test was applied to the difference scores to assess normality, guiding the selection of paired *t*-tests or Wilcoxon signed-rank tests as appropriate. Although non-significant trends toward increased diversity were noted post-intervention in the probiotic group, overall, the interventions did not result in consistent or significant modifications of global alpha diversity indices among children with ASD.

**Figure 10:**
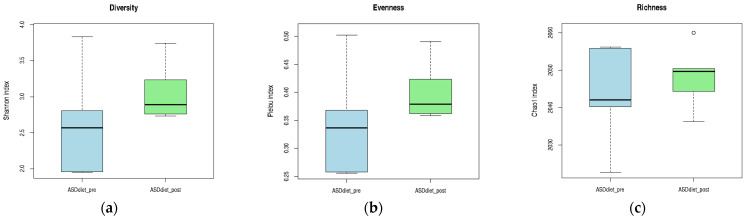
Alpha diversity indices in the ASD diet group before and after anti-inflammatory dietary intervention. (**a**) Shannon index (diversity), (**b**) Pielou index (evenness), and (**c**) Chao1 index (richness). Each panel shows the distribution of values before (blue) and after (green) the 12-week dietary intervention period.

Although no statistically significant differences were observed, all three alpha diversity metrics showed a tendency to increase following anti-inflammatory dietary intervention in the ASD_diet group. The median values for the Shannon (diversity) and Pielou (evenness) indices were higher POST-intervention, suggesting a more even and diverse microbial community. Similarly, the Chao1 richness index showed a slight increase in the central tendency, despite greater dispersion in PRE-intervention values. These trends may reflect a gradual modulation of the gut microbiota in response to dietary components, even if not strong enough to reach statistical significance within the sample size and time frame evaluated. The visual patterns presented in Figure 10 provide preliminary evidence of a positive shift in microbial ecology associated with nutritional intervention.

**Figure 11:**
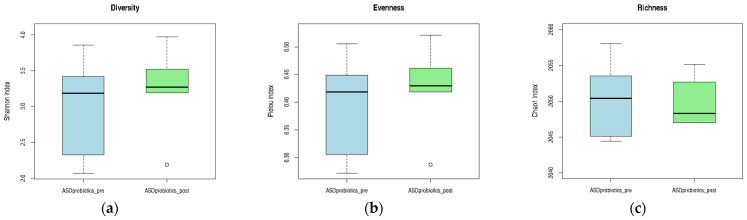
Alpha diversity indices in the ASD_probiotics group before and after probiotic intervention. (**a**) Shannon index (diversity), (**b**) Pielou index (evenness), and (**c**) Chao1 index (richness). Boxplots represent pre- (blue) and post-intervention (green) values after 12 weeks of probiotic supplementation.

In the ASD_probiotics group, within-subject comparison of alpha diversity indices revealed minimal changes in microbial diversity and evenness following the 12-week intervention. Both Shannon and Pielou indices remained relatively stable, with only slight increases in their median values. In contrast, the Chao1 richness index showed a tendency toward decreased richness after probiotic supplementation, as reflected by the lower median value POST-intervention. Although none of these trends reached statistical significance, the observed pattern suggests that probiotics exert a more selective influence on specific microbial populations rather than inducing broad changes in overall diversity. These trends are illustrated in Figure 11, which highlights the subtle shifts in the community structure associated with the intervention.

#### 2.4.2. Differential Abundance of Bacterial Genera Between PRE- and POST-Intervention Samples in ASD Subgroups

##### Differential Abundance of Bacterial Genera Before and After the Anti-Inflammatory Dietary Intervention

**Figure 12:**
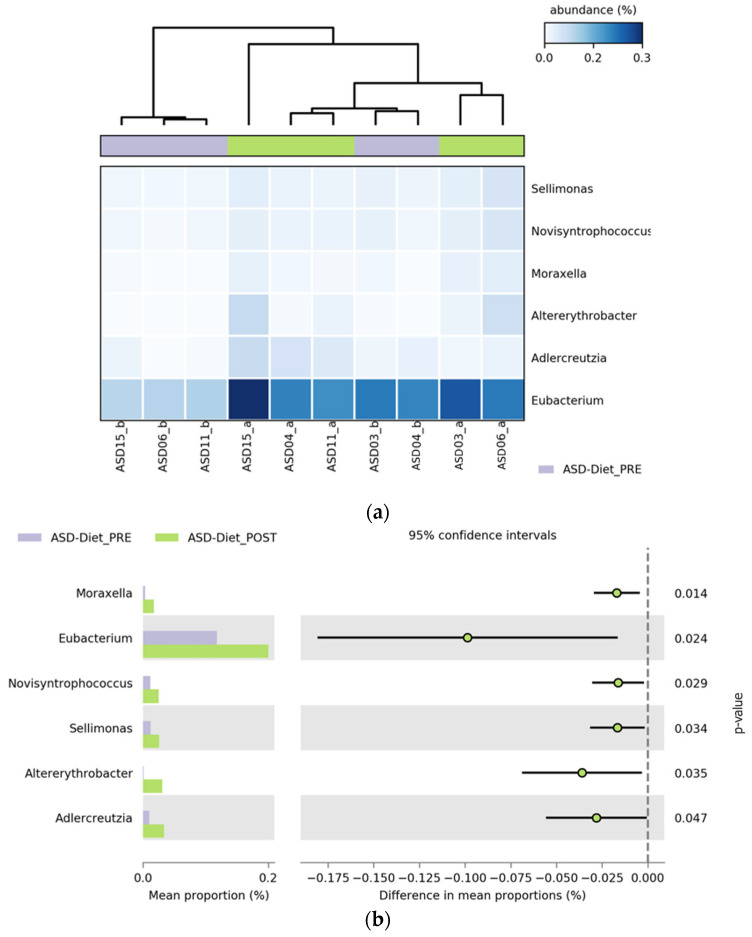
Differential abundance of bacterial genera in children with ASD before and after an anti-inflammatory dietary intervention. (**a**) Heatmap representing the relative abundance (%) of bacterial genera that changed significantly in ASD participants before and after the anti-inflammatory dietary intervention. Each column corresponds to a sample (PRE- or POST-intervention), and each row corresponds to a genus. The color gradient reflects relative abundance: lighter blue represents lower abundance, whereas darker blue indicates higher abundance levels. Relative abundance values were normalized across participants. (**b**) Extended error bar plot displaying differences in mean proportions, 95% confidence intervals, and *p*-values for each genus.

Following the anti-inflammatory dietary intervention, six bacterial genera showed a statistically significant increase in relative abundance in the ASD_diet group. These included *Moraxella*, *Eubacterium*, *Novisyntrophococcus*, *Sellimonas*, *Altererythrobacter*, and *Adlercreutzia*. The consistent upward trend across all genera suggested that the dietary strategy promoted the growth of specific microbial taxa, potentially contributing to a more balanced or metabolically favorable gut environment. Notably, *Moraxella* and *Eubacterium* exhibited the most pronounced increases, with FDR-corrected *p*-values of 0.014 and 0.024, respectively. As illustrated in Figure 12, these changes are visually represented by the shift in relative abundance in the heatmap (a) and confirmed by the extended error bar plot (b), which displays the direction and magnitude of differences, along with statistical significance.

**Figure 13:**
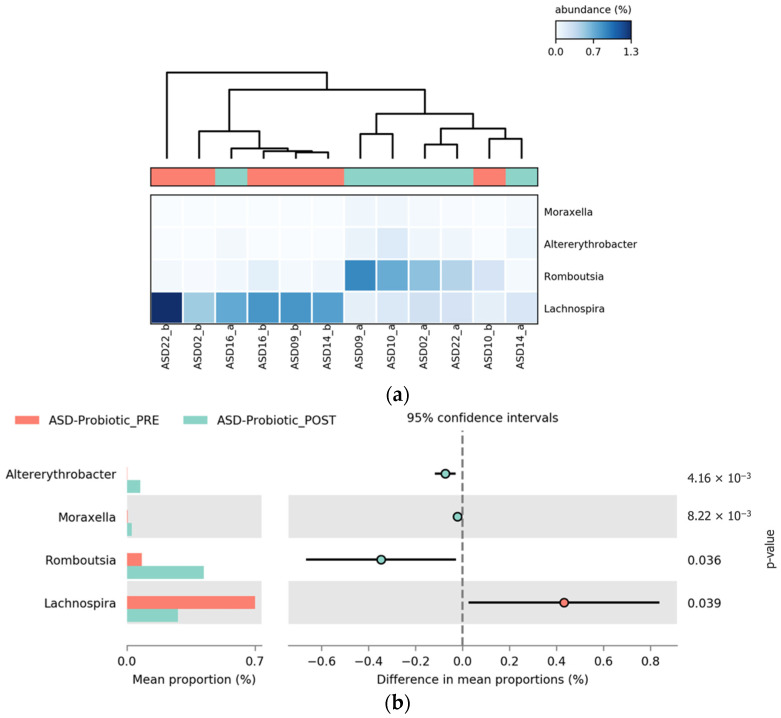
Differential abundance of bacterial genera in children with ASD before and after probiotic intervention. (**a**) Heatmap showing the relative abundance (%) of genera with significant differences between the PRE-(salmon) and POST-intervention (turquoise) samples. Columns represent pre- and post-intervention samples, and rows indicate bacterial genera. The color scale reflects relative abundance, where lighter blue indicates lower levels and darker blue higher abundance. Normalization was applied to allow for consistent interpretation across samples. (**b**) Extended error bar plot showing the differences in mean proportions, 95% confidence intervals, and *p*-values for each genus.

Probiotic intervention led to statistically significant changes in the abundance of the four bacterial genera within the ASD_probiotics group. Notably, *Romboutsia* exhibited a clear POST-intervention increase, whereas *Lachnospira* showed a significant decrease in relative abundance. These two genera demonstrated the largest shifts, suggesting their potential role in the microbial response to probiotic supplementation. In addition, moderate increases were observed in *Altererythrobacter* and *Moraxella*, which were statistically significant. These patterns, presented in Figure 13, are visualized in the heatmap (a) and the extended error bar plot (b) and may reflect targeted microbial modulation rather than broad changes in diversity. The opposing directions of change between *Lachnospira* and *Romboutsia* are particularly relevant and warrant further investigation regarding their functional implications in ASD-related gut dysbiosis.

### 2.5. Differential Immune–Microbial Correlation Patterns in ASD and TD Children

Analysis of immune–microbial associations revealed distinct network architectures in ASD patients compared to typically developing (TD) controls. Using a correlation threshold of |ρ| ≥ 0.5 (*p* < 0.05), significant cytokine–microbiota associations were identified in both groups. However, the composition and connectivity of these networks differ markedly.

In the ASD group, the network was characterized by IFN-γ as a major hub connected to multiple bacterial taxa, including *Blautia*, *Anaerostipes*, *Anaerobutyricum*, and *Lachnoclostridium*, suggesting an overrepresentation of inflammatory pathways linked to gut microbiota. Additional associations were observed between *Faecalibacterium* and IL-18, and between *Lachnospira* and RANTES, implicating SCFA-producing bacteria in immune modulation. Correlation strengths ranged from 0.44 to 0.77, indicating moderately strong interactions. Some positive correlations are present between *Bifidobacterium–IL-8* (ρ = 0.52) and *Faecalibacterium–IFN-γ* (ρ = 0.48), probably indicating a role in intestinal permeability and neuroinflammation, while *Roseburia* and *Prevotella* showed negative correlations with MIP-1α and MCP-1. Some of the negative correlations between MCP-1 and GRO-α in some genera (e.g., *Prevotella* and *Roseburia*) may suggest dysfunction in chemotactic pathways.

Conversely, the TD network displayed highly compact connectivity dominated by MCP-1 and IFN-γ, which were strongly correlated (ρ ≥ 0.9) with commensal genera, such as *Roseburia*, *Bifidobacterium*, and *Parabacteroides*. This pattern suggests a more stable immune–microbial relationship in neurotypical children, possibly reflecting balanced pro- and anti-inflammatory signaling. In the present study, stronger correlations were observed, highlighting the positive associations between fermenting bacteria (*Faecalibacterium*, *Roseburia*), IL-8 (ρ = 0.72), and IFN-γ (ρ = 0.65). Robust correlations were also found for *Bacteroides-RANTES* (ρ = 0.58) and *Prevotella-MCP-1* (ρ = 0.60). This may indicate that the microbiome is more capable of modulating physiological inflammation.

**Figure 14:**
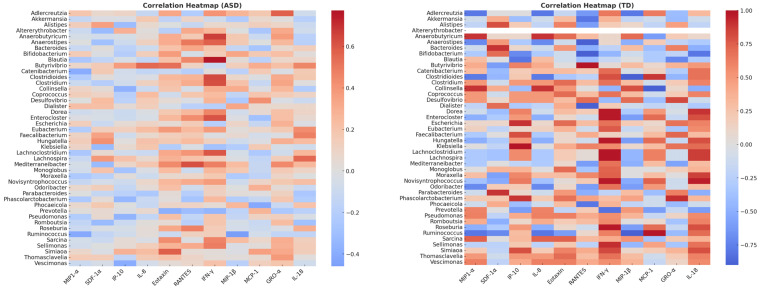
Correlations between gut bacterial genera and plasma cytokines in children with ASD and typically developing (TD) controls. Heatmaps depict Spearman correlation coefficients between the relative abundances of gut bacterial genera (rows) and plasma cytokine/chemokine concentrations (columns) in the ASD (**left**) and TD (**right**) groups. Positive correlations are shown in red and negative correlations are shown in blue. Stronger correlations (|ρ| ≥ 0.5) indicate more consistent immune–microbial interactions. Differences in correlation patterns suggest disrupted immune–microbiota networks in ASD compared to TD, reflecting potential dysbiosis and immune dysregulation.

Heatmap visualization confirmed these differences: ASD profiles exhibited broader immune connectivity with pro-inflammatory cytokines, whereas TD networks were more selective, but stronger in magnitude (Figure 14).

### 2.6. Construction of Immuno-Microbial Networks

To identify possible immuno-microbial networks, metagenomic data and plasma levels of cytokines in children with autism spectrum disorder (ASD) who received an anti-inflammatory diet (*NeuroGutPlus*) and probiotic intervention for 12 weeks were analyzed.

**Figure 15:**
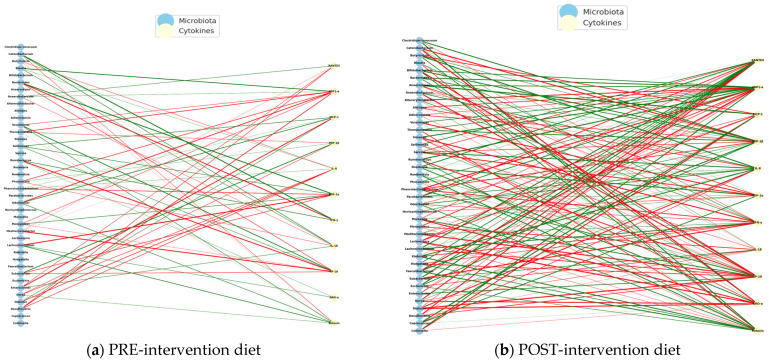
Microbiota–cytokine interaction networks before and after anti-inflammatory diet intervention. The bipartite networks showed significant correlations between gut microbial genera and plasma cytokines in children with ASD subjected to an anti-inflammatory dietary intervention. Blue nodes represent bacterial genera; yellow nodes represent cytokines. Node size is proportional to degree centrality (nodes with more significant connections are larger). Edges represent significant correlations (Spearman’s ρ, *p* < 0.05); thicker edges indicate stronger correlations. (**a**) PRE-intervention and (**b**) POST-intervention. Edge colors indicate the correlation direction: *green* indicates positive associations, and *red* indicates negative associations. Node size is proportional to degree centrality, with larger nodes representing taxa or cytokines with more significant connections in the network. Post-intervention, the network shows a reduction in negative associations and an increase in positive links, suggesting a shift toward a more balanced immune–microbial interaction pattern.

Spearman correlations were calculated between bacterial abundance and cytokine levels at the time of PRE- and POST- intervention. On the left are the bacteria and cytokine nodes on the right, with green links indicating positive correlations and red links indicating negative correlations. Structural changes and key nodes (immune hubs) were analyzed. Figure 15 presents the PRE-diet intervention interaction networks with mostly negative correlations between bacteria and pro-inflammatory cytokines.

The POST-intervention analysis increased positive correlations, possibly reflecting a pro-inflammatory regulation controlled by diet intervention.

The PRE-intervention immuno-microbial network showed a high proportion of negative correlations, where unfavorable interactions between bacteria and inflammatory cytokines predominate. On the other hand, some connections between IFN-γ and MIP-1β stand out, indicating a pro-inflammatory environment. Some of the negatively connected genera include *Escherichia*, *Clostridium*, *Klebsiella*, *Prevotella*, and *Odoribacter*, suggesting dysbiosis associated with an altered immune profile.

The POST- intervention shows a significant reorganization with the entry of new central cytokines (*IL-18* and *IL-8*), suggesting a modulation of the systemic inflammatory profile. The reduction of nodes such as *MCP-1* and POST-intervention *RANTES* could reflect lower cellular chemotaxis and lower basal immune activation, compatible with a diet-induced immunoregulatory effect. Table 6 shows the interaction networks between the gut microbiota and the plasma cytokine profile in children with ASD before (left) and after (right) receiving an anti-inflammatory diet (*NeuroGutPlus*).

The observed transition from a disconnected and negative immuno-microbial network (PRE) to a more integrated and positive network (POST) indicates a relevant functional impact of an anti-inflammatory diet in children with ASD. Positive POST-intervention correlations, especially with cytokines such as *IL-8*, *MIP-1β*, and *SDF-1α*, could reflect the inflammatory resolution and tissue repair processes and functions attributed to these cytokines in neuroimmune contexts [28].

Strengthening partnerships with genera such as *Blautia*, *Ruminococcus*, and *Mediterraneibacter*, which produce short-chain fatty acids (SCFAs) such as butyrate, supports their role as key mediators of the gut–brain axis, modulating the gut barrier, microglia, and neurotransmitter production [55].

**Figure 16:**
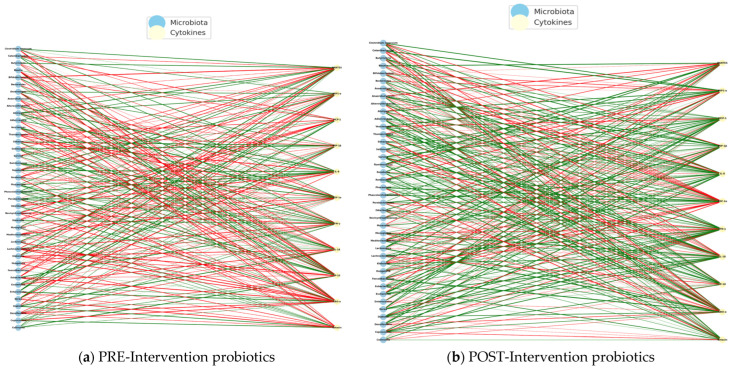
Microbiota–cytokine interaction networks before and after probiotic intervention. The bipartite networks showed significant correlations between gut microbial genera and plasma cytokines in children with ASD who were subjected to probiotic intervention. Blue nodes represent bacterial genera; yellow nodes represent cytokines. Node size is proportional to degree centrality (nodes with more significant connections are larger). Edges represent significant correlations (Spearman’s ρ, *p* < 0.05); thicker edges indicate stronger correlations. (**a**) PRE-intervention and (**b**) POST-intervention. Edge colors indicate the correlation direction: *green* indicates positive associations, and *red* indicates negative associations. Node size is proportional to degree centrality, with larger nodes representing taxa or cytokines with more significant connections in the network. POST-intervention, the network shows a reduction in negative associations and an increase in positive links, suggesting a shift toward a more balanced immune–microbial interaction pattern.

Regarding the correlations obtained from the intervention with probiotics in the study group, we identified negative correlations in the PE intervention, indicating that the greater the abundance of certain bacterial genera, the lower the levels of inflammatory cytokines, such as IL-8, IP-10, or M1P1-α. This pattern may represent an attempt at natural immune compensation, in which certain bacteria act to dampen the underlying systemic inflammation. In this state, the microbiota could be in an immunological containment mode, but without overall effectiveness, given the typical imbalance in ASD (Figure 16a).

In the POST-intervention period, a predominance of positive correlations was identified, which suggests that the abundance of certain bacterial genera is associated with an increase in specific cytokines, many of which are involved in the regulatory immune response, such as *MIP1-α*, *SDF-1α*, *IL-8*, and *Eotaxin*. This change in direction suggests that probiotics not only restore beneficial bacterial populations, but also reprogram the microbiota–immunity network towards positive cooperation, where the microbiota activates functional, non-inflammatory, and immune responses (Figure 16b).

It could be inferred that the intervention with probiotics not only stabilized the immuno-microbial network but also reversed the profile of bacteria–cytokine correlations, indicating a favorable immune reprogramming, in which the intestinal microbiota promotes a regulated immune activation, contributing to a more competent and less pathological response.

During the PRE-intervention, the network showed a significant number of negative correlations between bacteria and inflammatory cytokines, suggesting a state of defensive dysbiosis. After intervention with probiotics, a reconfiguration of the network was observed, where positive correlations with regulatory cytokines predominated, and an increased connectivity of beneficial bacteria known for their role in the production of butyrate and SCFAs.

The functional shift in the direction of correlations (from negative to positive) suggests that probiotics not only restore specific bacterial communities but could also modulate their functional relationship with the immune system. This pattern could indicate that the microbiome stimulates tolerant and effective immune responses rather than chronic inflammatory ones [23,56].

POST-intervention, central immune nodes such as *SDF-1α* and *Eotaxin* are linked to tissue regeneration and cell migration. Bacteria such as *Blautia*, *Roseburia*, and *Ruminococcus* are associated with a reduction in intestinal permeability and neuroinflammation in animal and human models of ASD. The findings presented here reinforce the notion that probiotics can induce a state of regulatory immunocompetence, facilitating effective communication between the microbiota and immune system in children with ASD. This reconfiguration may be related to the clinical improvements observed in the gastrointestinal, behavioral, and neurocognitive symptoms in children with ASD.

**Figure 17:**
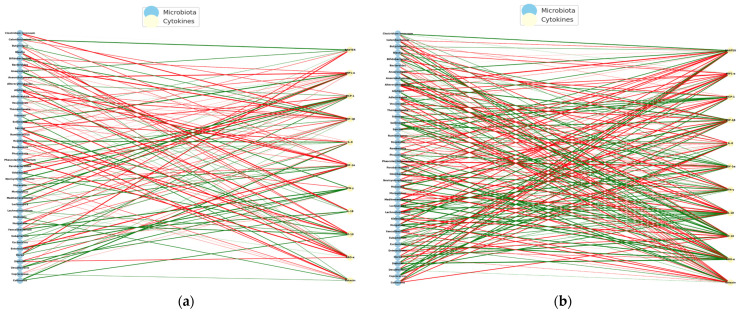
Immune–microbiota interaction networks in the control group without dietary or probiotic intervention (PRE vs. POST 12 weeks). (**a**) Immune–microbiota correlation network before the 12 weeks. (**b**) Immune–microbiota correlation network after the 12 weeks. Bipartite networks illustrating correlations (Spearman, |ρ| ≥ 0.5, *p* < 0.05) between gut microbial genera (blue nodes) and plasma cytokines (yellow nodes) in the control group before (left) and 12 weeks after (right) without dietary or probiotic intervention. Green edges indicate positive correlations, whereas red edges represent negative correlations. Lower network density and reduced connectivity were observed compared to the intervention groups (diet or probiotics). Persistent hubs, such as IL-18 and IFN-γ, suggest a basal pro-inflammatory state typically associated with ASD and are possibly linked to chronic dysbiosis.

In the control group, without dietary or probiotic intervention with PRE and POST measurements at 12 weeks, there were fewer interactions with a greater diversity of nodes. The cytokines *IL-18* and *IFN-γ* may reflect a basal inflammatory background typical of ASD, possibly linked to persistent dysbiosis (Figure 17).

A greater number of positive correlations were identified in the immune–microbiota correlation before 12 weeks. After 12 weeks, an increase in negative correlations was observed, especially between genera such as *Escherichia*, *Odoribacter*, and *Blautia* and cytokines such as IL-8 and IFN-γ. Bacterial genera, such as *Bacteroides*, *Alistipes*, *Prevotella*, *and Faecalibacterium*, showed multiple correlations both before and after 12 weeks. The increase in negative interactions between proinflammatory cytokines and commensal bacteria (*Ruminococcus*, *Lachnospira*) after 12 weeks may reflect endogenous immunological processes of self-regulation or mild natural dysbiosis. Finally, the absence of important structural changes in the network suggests that, without nutritional or pharmacological intervention, the immune–microbial system in children with ASD maintains a basal, possibly dysfunctional, but stable interaction in the short term. Studies such as that of Kang et al. observed that without intervention, bacterial and cytokine profiles in children with ASD tend to be maintained for 8–12 weeks, although with high interindividual variability [57].

This comparison revealed that the control group without intervention maintained a relatively stable immuno-microbial structure but with a tendency to increase negative correlations over time. This could indicate a progressive dysfunction of the gut–immunity axis in ASD in the absence of intervention, which reinforces the need for specific therapeutic strategies.

## 3. Discussion

This study provides the first comprehensive characterization of the gut microbiota in Colombian children with autism spectrum disorder (ASD) using shotgun metagenomic sequencing while simultaneously evaluating the modulatory effects of an anti-inflammatory diet and a probiotic formulation. Our findings reveal distinct microbial and immune perturbations in ASD, the exacerbating role of functional gastrointestinal disorders (FGIDs), and the ability of targeted interventions to reorganize immune–microbial networks toward cooperative and regulatory configurations.

At baseline, ASD children exhibited a higher *Firmicutes*/*Bacteroidetes (F*/*B)* ratio and enrichment of pro-inflammatory taxa such as *Clostridioides*, *Alistipes*, *Thomasclavelia*, and *Coprococcus* alongside reduced abundance of short-chain fatty acid (SCFA)-producing genera including *Faecalibacterium* and *Roseburia*. These compositional shifts align with global reports linking ASD to dysbiosis and impaired SCFA biosynthesis pathways [58,59], which are critical for gut barrier integrity and immune regulation. Overrepresented genera, particularly *Alistipes* and *Clostridioides*, have been implicated in lipopolysaccharide (LPS) biosynthesis and systemic immune activation via TLR4/MyD88/NF-κB signaling, reinforcing the pro-inflammatory gut milieu observed in ASD cohorts worldwide [60,61,62]. The depletion of butyrate-producing taxa supports the hypothesis of a disrupted gut–immune–brain axis, as SCFAs, such as butyrate, modulate regulatory T-cell differentiation, epithelial integrity, and microglial homeostasis [63,64].

Network analyses further demonstrated that ASD immune–microbial architectures were highly fragmented, with dominant hubs involving IFN-γ and MCP-1—canonical markers of Th1-driven inflammation and chemokine-mediated leukocyte recruitment. Conversely, neurotypical (TD) networks exhibited more integrated structures, centered on MCP-1, IL-8, and RANTES, and maintained strong positive associations with commensal taxa, such as *Bacteroides* and *Bifidobacterium*. These contrasting topologies suggest that ASD pathophysiology involves not only compositional dysbiosis but also the collapse of functional immune–microbial connectivity, potentially driven by diminished metabolite-mediated immunoregulation and increased microbial pro-inflammatory signaling [63,64,65].

FGIDs have emerged as a critical comorbidity that amplifies microbial instability and immune disruption in ASD patients. Children with ASD + FGID exhibited a significant reduction in species richness (lower Chao1 index) and an enrichment of pro-inflammatory genera such as *Parabacteroides* and *Alistipes*, coupled with highly fragmented immune–microbial networks. These findings support prior evidence linking gastrointestinal dysfunction to increased systemic inflammation, compromised mucosal integrity, and worsened behavioral outcomes in ASD [65,66]. Notably, even TD children with FGIDs showed depletion of SCFA-producing taxa (*Coprococcus* and *Anaerobutyricum*), underscoring that gastrointestinal symptomatology, regardless of neurodevelopmental status, undermines microbiome–immune synergy. However, the compounded impact observed in ASD suggests a gene–microbiota–environment interplay amplifying systemic inflammation and neurodevelopmental vulnerability [67].

Both microbiota-targeted interventions induced structural reorganization of immune–microbial networks. The 12-week anti-inflammatory diet was designed using regionally accessible polyphenol-rich and fiber-dense foods and enriched SCFA-associated taxa such as *Eubacterium*, *Adlercreutzia*, and *Moraxella*, while reducing negative bacteria–cytokine correlations. POST-intervention networks were positively associated with IL-8, SDF-1α, and MIP-1βcytokines implicated in tissue repair and controlled chemotaxis [68,69]. These findings align with clinical evidence supporting Mediterranean-style diets and polyphenol supplementation as modulators of oxidative stress, immune tolerance, and microbiota composition in ASD and related neurodevelopmental disorders [70,71].

Probiotic supplementation produced a distinct yet complementary effect, characterized by selective enrichment of *Romboutsia* and a decline in *Lachnospira*, along with a functional transition from antagonistic immune–microbial interactions to cooperative architectures dominated by SCFA producers (*Blautia*, *Roseburia*) and regulatory cytokines (Eotaxin, SDF-1α). This realignment supports mechanistic models proposing that probiotics exert strain-specific effects via metabolite-mediated pathways, fostering Treg differentiation and dampening Th1-driven inflammation [72,73]. These findings resonate with meta-analyses and controlled trials reporting that probiotics alleviate gastrointestinal symptoms and behavioral disturbances in ASD, although taxonomic restoration remains partial [74,75]. Collectively, the diet and probiotic arms illustrate complementary strategies: diets broadly reshape microbial metabolic landscapes through substrate-driven mechanisms, whereas probiotics provide precise modulation by introducing keystone taxa or functions.

Our results underscore the gut microbiome as both a biomarker and therapeutic target in ASD, supporting the development of precision-based strategies that combine nutritional and microbial therapeutics. Shotgun metagenomics enables high-resolution profiling, capturing taxonomic and functional shifts, and within-group variability that would likely remain undetected by 16S-based approaches. Coupled with cytokine mapping, this multi-omics framework facilitates the design of personalized interventions based on baseline microbial and immunological signatures [61,76].

Nevertheless, certain limitations of this study warrant consideration. The relatively small sample size within the intervention arms reduces statistical power and generalizability. The 12-week intervention window, which was sufficient to induce structural network reconfiguration, may have been too short to elicit robust changes in alpha diversity or sustained clinical improvements. Moreover, the absence of metabolomic validation (e.g., SCFA quantification) constrains mechanistic inference regarding microbial metabolite-driven immune modulation. These limitations are pervasive in ASD microbiome research, including heterogeneity in study design and short follow-up durations. Future studies should address these gaps by implementing larger, adequately powered, randomized controlled trials that incorporate multi-omics layers and extended observation periods. Additionally, combined interventions leveraging diet, probiotics, and pharmacological agents should be explored to harness the potential synergistic benefits.

Importantly, although metabolomic data were not collected in this study, the enrichment of several genera with established short-chain fatty acid (SCFA)-producing capacity—including *Eubacterium*, *Adlercreutzia*, *Blautia*, and *Roseburia*—provides indirect evidence for a potential increase in microbial metabolic activity associated with immune regulation. SCFAs, particularly butyrate and propionate, are known to reinforce epithelial barrier integrity, promote the differentiation of regulatory T cells, and attenuate pro-inflammatory cytokine production. The appearance of these taxa in post-intervention networks therefore suggests a functional shift toward a more immunoregulatory microbial environment. Nevertheless, targeted metabolomic validation (e.g., direct quantification of SCFAs) will be crucial in future studies to confirm these mechanistic links and strengthen causal inference.

In the control group, without dietary or probiotic intervention, the immune–microbial network showed minimal restructuring over the 12-week follow-up, which we initially described as “relatively stable”. However, we acknowledge that this group exhibited a statistically significant reduction in Chao1 richness (*p* < 0.05), indicating a measurable decline in microbial richness. This reduction may reflect a natural progression of dysbiosis in children with ASD over time, even in the absence of targeted intervention, consistent with previous reports describing microbiota instability in ASD cohorts [77,78]. Thus, while the overall network topology remained largely unchanged, the quantitative decrease in richness highlights an underlying trend of microbiota deterioration that may contribute to the persistence of immune dysregulation in this population.

Since our trial enrolled only children with high-functioning ASD (DSM-5 Level I), caution is warranted when extrapolating to children with greater support needs (Levels II–III) or co-occurring intellectual disability. These groups tend to show a higher prevalence of gastrointestinal symptoms, more pronounced food selectivity and micronutrient deficiencies, and greater use of psychotropic medications [79,80,81,82]—all factors associated with reduced microbial diversity and altered community structure. Such baseline differences may amplify or reshape dysbiosis and could modify both the magnitude and the direction of microbiota–immune network changes in response to diet or probiotics. Future work should therefore (i) recruit across the full severity spectrum and stratify by intellectual disability; (ii) record and adjust for GI comorbidities, antibiotic and psychotropic exposure, and dietary therapy participation; and (iii) integrate metabolomics to validate mechanism. These steps will better define which ASD phenotypes derive the greatest benefit from microbiota-targeted nutrition.

In conclusion, Colombian children with ASD exhibited distinct microbial dysbiosis, immune–microbial network fragmentation, and heightened vulnerability to FGIDs. Both anti-inflammatory diet and probiotic interventions promoted structural reorganization of immune–microbial networks toward cooperative and regulatory configurations, despite modest effects on alpha diversity. These findings highlight the therapeutic potential of microbiota-targeted strategies as adjunctive interventions in ASD and provide a conceptual framework for developing precise nutrition and microbial therapeutics informed by multi-omics biomarkers.

## 4. Materials and Methods

### 4.1. Study Design

This study was conducted in two phases. The first phase involved a descriptive cross-sectional analysis of the gut microbiota through metagenomic profiling in children diagnosed with autism spectrum disorder ASD (n = 23) compared to neurotypical controls (TD, n = 7). The second phase was a trial with an experimental design involving 18 ASD participants from phase one. These children were assigned to three parallel groups (n = 6 each): two intervention arms, one receiving a probiotic formulation and the other an anti-inflammatory diet (*NeuroGutPlus*), and a non-intervention control group. “*The anti-inflammatory dietary intervention, hereafter referred to as “NeuroGutPlus” was specifically formulated for this study using regionally accessible, polyphenol-rich, and fiber-dense foods. NeuroGutPlus is not a trade name but a label used to designate the study’s anti-inflammatory diet protocol*”.

Shotgun metagenomic sequencing of fecal samples was performed at baseline and after the 12-week intervention period.

### 4.2. Participants and Eligibility Criteria

Participants included boys and girls aged between 5 and 17 years with a confirmed clinical diagnosis of ASD based on the criteria established by pediatric neurology or child psychiatry professionals in accordance with the national clinical protocol for ASD diagnosis and care (MinSalud, 2015 [7]). Only children with high-functioning autism (Level I, DSM-5) and active affiliation with the Colombian General System of Social Health Insurance (SGSSS) were included. Informed consent was obtained from the parents or legal guardians along with a child assent. Participants were recruited from therapeutic support institutions and ASD networks in Colombia’s Eje Cafetero region, including the Instituto para el Desarrollo Integral del Niño Autista (DINA) and Red TEA Caldas. Caregivers were actively engaged through informational sessions, opportunities for questions, and continuous communication to ensure culturally sensitive and ethically appropriate participation.

Exclusion Criteria: Children were excluded if they had neurological, neuromuscular, or psychiatric conditions other than ASD; diagnosed food allergies (e.g., cow’s milk protein), celiac disease, or other immune-related disorders; or used antibiotics, antifungals, corticosteroids, or immunosuppressive agents within three months prior to sample collection or intervention onset.

### 4.3. Metagenomic Analysis of Gut Microbiota

#### 4.3.1. Sample Collection and Preservation

Fecal samples were collected at the participants’ homes under standardized procedures following a detailed protocol provided to caregivers. Samples were immediately stored at −18 °C and transported under cryopreserved conditions using insulated containers with ice packs to maintain cold chain integrity. Upon arrival, the samples were stored at −40 °C until further processing.

#### 4.3.2. DNA Extraction and Quality Control

Total microbial DNA was extracted using the QIAamp^®^ Fast DNA Stool Mini Kit (Qiagen, Hilden, Germany) following the manufacturer’s protocol and optimized for maximum yield and purity. Two technical replicates per sample were processed, and the replicate with the best purity ratio (A260/A280 = 1.7–1.9) was selected for downstream applications. DNA purity and concentration were evaluated using a BioTek EPOCH™ Microplate Spectrophotometer and Gen5™ software, version 3.16.10 (Agilent Technologies, Santa Clara, CA, USA). The extracted DNA samples were stored at −40 °C until sequencing.

#### 4.3.3. Library Preparation and Sequencing

Shotgun metagenomic sequencing was performed using the Illumina NextSeq™ 2000 platform (Illumina, San Diego, CA, USA) with paired-end 150 bp reads and a total cycle count of 300, employing the XLEAP-SBS™ Reagent Kit P4 (300 cycles Illumina, San Diego, CA, USA). Library preparation followed the standard protocols for whole-genome sequencing with fragmentation, end-repair, adapter ligation, and PCR amplification.

#### 4.3.4. Bioinformatics Processing and Taxonomic Profiling

Raw sequencing reads were preprocessed using FastP v.1.01 for adapter trimming, quality filtering (Q > 30), and removal of low-complexity sequences. Taxonomic classification was conducted using Kraken2 v.2.0.7, employing a custom database based on the NCBI for Biotechnology Information RefSeq complete genome set for bacteria, archaea, and viruses. Downstream analyses included the computation of alpha and beta diversity indices using the vegan, fossil, ecolTest, and microbiome R packages. Community composition, taxonomic abundance, and intergroup comparisons were assessed using STAMP (v2.1.3) and RStudio (v4.2.1).

### 4.4. Cytokine Profiling and Immune–Microbiota Correlation Analysis

Plasma samples were collected from children with ASD before and after the 12-week intervention period to evaluate systemic immune responses. Cytokine concentrations were quantified using the ProcartaPlex™ Human Th1/Th2 & Chemokine Panel 20plex (Catalog No. EPX200-12173-901; 96 tests; Thermo Fisher Scientific, Waltham, MA, USA), based on Luminex xMAP^®^ technology, using a MAGPIX^®^ instrument with xPONENT^®^ software, version 4.2 (Thermo Fisher Scientific, Waltham, MA, USA). From the full panel, 11 cytokines were selected for analysis based on their detectability and biological relevance: MIP-1α, SDF-1α, IP-10, IL-8, Eotaxin, RANTES, IFN-*γ*, MIP-1β, MCP-1, GRO-α, and IL-18. The assays were performed in duplicate.

To explore the functional interactions between the gut microbiota and host immune response, Spearman correlation coefficients (ρ) were computed between the relative abundance of bacterial genera (obtained from shotgun metagenomic sequencing) and the plasma levels of the 11 cytokines, both PRE- and POST-intervention. Only correlations with |ρ| ≥ 0.5 and *p* < 0.05 were retained to construct bipartite immune–microbial networks. Nodes represent bacterial genera and cytokines, while edges are colored based on the direction of correlation (green for positive, red for negative). Changes in network topology, hub cytokines, and centrality were analyzed to identify potential immunoregulatory patterns modulated by dietary or probiotic treatments.

### 4.5. Statistical Analysis

Normality of distributions was assessed with the Shapiro–Wilk test, and homogeneity of variances with Levene’s test. Depending on whether assumptions were met, comparisons between two groups were performed using either independent-sample *t*-tests (parametric) or Mann–Whitney *U* tests (non-parametric). For comparisons across more than two groups, one-way ANOVA was applied when assumptions were satisfied, and the Kruskal–Wallis test was used otherwise. Post hoc analyses included Bonferroni or Dunn’s corrections as appropriate.

Alpha diversity indices (Shannon, Pielou, and Chao1) were calculated in QIIME2 and compared across groups using Kruskal–Wallis or Mann–Whitney U tests, according to distributional assumptions. Taxonomic differences in relative abundance were analyzed using STAMP (v2.1.3), applying Welch’s *t*-tests or ANOVA, with Benjamini–Hochberg false discovery rate (FDR) correction for multiple testing. Cytokine concentrations in plasma were evaluated with GraphPad Prism (version 9.5.0) using paired or unpaired *t*-tests, repeated-measures ANOVA, or their non-parametric equivalents depending on distribution. Correlation analyses between microbial taxa and cytokines were performed using Spearman’s rank correlation (ρ). Correlation matrices and network construction were performed in R (version 4.3.2; R Foundation for Statistical Computing, Vienna, Austria) and Python (version 3.11; Python Software Foundation), and network visualizations were produced in Cytoscape (version 3.10.3).

All tests were two-tailed, and *p* < 0.05 was considered statistically significant unless otherwise indicated.

## 5. Conclusions

Intervention with an anti-inflammatory diet produced evident changes in the immuno-microbial network, highlighting a decrease in negative interactions and an increase in positive connectivity with beneficial genera, shifting the proinflammatory central axis towards an immunoregulatory profile. These visual findings support the hypothesis that an anti-inflammatory diet can restore protective immuno-microbial interactions in children with ASD.

Multistrain probiotic supplementation in children with ASD leads to profound modulation of immuno-microbial networks, characterized by a reversal in the directionality of correlations and greater functional integration. This finding supports the use of microbiological strategies as therapeutic adjuvants for the treatment of ASD, with measurable and potentially clinically relevant immunoregulatory effects.

### Limitations and Future Directions

While the findings presented here are encouraging, it is important to recognize several ethical, cultural, and technical limitations that may influence both interpretation and applicability. Ethically, implementing dietary and probiotic interventions in children calls for particular care—especially when considering informed consent, continuous safety monitoring, and the possibility of unexpected metabolic or immune-related effects. Working with pediatric populations requires not only scientific rigor but also empathy and respect for caregivers’ concerns and the children’s developmental needs. Cultural aspects also play a key role: food preferences, access to certain ingredients, and family beliefs about nutrition can significantly affect adherence to dietary protocols and, ultimately, the success of the intervention—especially in low- and middle-income settings.

Technically, the relatively small sample size and short intervention period (12 weeks) may have limited the ability to detect more subtle or long-term microbiota changes, particularly at the diversity level. Additionally, including only high-functioning children with ASD restricts (DSM-5 Level I) the generalization of our findings to broader ASD populations. Finally, although shotgun metagenomics provided valuable taxonomic insights, the lack of functional validation—such as targeted metabolomics or metatranscriptomics—limits our understanding of how microbial changes translate into immune modulation. Near-future studies would benefit from more extended follow-up periods, more diverse and representative cohorts, and the integration of multi-omics approaches that move beyond taxonomic profiling. Combining tools like targeted metabolomics and metatranscriptomics can help uncover causal relationships and deepen our understanding of how microbial changes impact host physiology—ultimately paving the way for more personalized and effective microbiota-based therapies.

## Figures and Tables

**Table 1:** Sociodemographic characteristics of study participants.

Variable	ASD (n = 23)	TD (n = 7)
**Sex, n (%)**		
Male	20 (86.9%)	4 (57.1%)
Female	3 (13.1%)	3 (42.9%)
**Presence of FGIDs, n (%)**		
Yes	15 (65.2%)	4 (57.1%)
No	8 (34.8%)	3 (42.9%)
**Age (years)**		
Mean	10.3	12.1
Range	6–16	10–15
**Height-for-age, n (%)**		
Adequate	17 (73.9%)	7 (100%)
At risk of delay	5 (21.7%)	0 (0%)
Low	1 (4.3%)	0 (0%)
**BMI-for-age, n (%)**		
Adequate	9 (39.1%)	4 (57.1%)
At risk of thinness	5 (21.7%)	0 (0%)
Thinness	1 (4.3%)	0 (0%)
Overweight	5 (21.7%)	1 (14.3%)
Obesity	3 (13.0%)	2 (28.6%)

**Table 2:** Sociodemographic characteristics of children with ASD by intervention group.

Variable	ASD Control (n = 6)	ASD Diet (n = 5)	ASD Probiotics (n = 6)
**Sex, n (%)**			
Male	4 (66.7%)	4 (80.0%)	6 (100%)
Female	2 (33.3%)	1 (20.0%)	0 (0%)
**Presence of FGIDs, n (%)**			
Yes	5 (83.3%)	2 (40.0%)	6 (100%)
No	1 (16.7%)	3 (60.0%)	0 (0%)
**Age (years)**			
Mean	10.7	10.8	10
Range	6–16	8–14	6–14
**Height-for-age, n (%)**			
Adequate	3 (50.0%)	5 (100%)	5 (83.3%)
At risk of delay	3 (50.0%)	0 (0%)	1 (16.7%)
Low	0 (0%)	0 (0%)	0 (0%)
**BMI-for-age, n (%)**			
Adequate	4 (66.7%)	2 (40.0%)	5 (83.3%)
At risk of thinness	2 (33.3%)	0 (0%)	0 (0%)
Thinness	0 (0%)	2 (40.0%)	1 (16.7%)
Overweight	0 (0%)	1 (20.0%)	0 (0%)
Obesity	0 (0%)	0 (0%)	0 (0%)

**Table 3:** Comparative statistical analysis of alpha diversity, evenness, and richness indices between children with ASD and TD.

Index	Shapiro–Wilk	Levene Test	*t*-Test	Mann–Whitney U
	*p*-Value	Pr (>F)	*p*-Value	*p*-Value
**Shannon**				
TD	0.5801	0.3718	0.2129	N/A
ASD	0.3204			
**Pielou**				
TD	0.58	0.3722	0.2118	N/A
ASD	0.3221			
**Chao1**				
TD	0.5241	N/A	N/A	0.2023
ASD	0.005335			

Statistical comparisons were performed between unpaired groups based on the results of the Shapiro–Wilk and Levene’s tests. Depending on the assumption of normality and homogeneity of variances, either a *t*-test or Mann–Whitney U test was applied.

**Table 4:** Comparative statistical analysis of alpha diversity, evenness, and richness indices in the ASD and TD subgroups stratified by the presence or absence of FGIDs.

Group Comparison	Index	Shapiro–Wilk *p*-Value		Levene Test	*t*-Test	Mann–Whitney U
				Pr (>F)	*p*-Value	*p*-Value
**TD vs. ASD**	**Shannon**	TD	0.5443	0.1411	0.9102	N/A
		ASD	0.7975			
	**Pielou**	TD	0.5467	0.1412	0.9108	N/A
		ASD	0.7946			
	**Chao1**	TD	0.3952	0.7223	0.05899	N/A
		ASD	0.7703			
**TD vs. TD_FGIDs**	**Shannon**	TD	0.5443	0.4852	0.1076	N/A
		TD_FGIDs	0.6675			
	**Pielou**	TD	0.5467	0.4856	0.1075	N/A
		TD_FGIDs	0.6661			
	**Chao1**	TD	0.3952	N/A	N/A	0.05714
		TD_FGIDs	0.0085			
**TD vs. ASD_FGIDs**	**Shannon**	TD	0.5443	0.1369	0.9309	N/A
		ASD_FGIDs	0.4672			
	**Pielou**	TD	0.5467	0.137	0.9325	N/A
		ASD_FGIDs	0.4643			
	**Chao1**	TD	0.3952	N/A	N/A	0.03302 *
		ASD_FGIDs	0.0115			
**TD_FGIDs vs.** **ASD_FGIDs**	**Shannon**	TD_FGIDs	0.6675	0.4136	0.09139	N/A
		ASD_FGIDs	0.4672			
	**Pielou**	TD_FGIDs	0.6661	0.4135	0.09088	N/A
		ASD_FGIDs	0.4643			
	**Chao1**	TD_FGIDs	0.0085	N/A	N/A	0.9635
		ASD_FGIDs	0.0115			

Normality was assessed using Shapiro–Wilk and homogeneity of variances using Levene’s test. Depending on data distribution, either unpaired *t*-test or Mann–Whitney U test was applied. A statistically significant reduction in Chao1 richness was observed in ASD children with FGIDs compared to TD controls (*p* = 0.03302 *).

**Table 5:** Comparative analysis of alpha diversity, evenness, and richness indices in children with ASD before and after microbiota-targeted intervention.

Group	Index	Shapiro-Wilk *p*-Value	*t*-Test *p*-Value
**ASD_control** **Pre vs. Post**	Shannon	0.1777	0.08734
	Pielou	0.175	0.0872
	Chao1	0.3645	0.03184 *
**ASD_diet** **Pre vs. Post**	Shannon	0.1246	0.1246
	Pielou	0.1249	0.2041
	Chao1	0.8267	0.1998
**ASD_probiotics** **Pre vs. Post**	Shannon	0.3359	0.6285
	Pielou	0.3337	0.6275
	Chao1	0.09577	0.5361

Comparative analysis of alpha diversity (Shannon), evenness (Pielou), and richness (Chao1) indices in children with ASD before and after microbiota-targeted interventions. Data are shown as paired comparisons within each group (ASD_control, ASD_diet, ASD_probiotics). Normality of the differences was assessed using the Shapiro–Wilk test, followed by paired *t*-test. A statistically significant reduction in Chao1 richness was observed in the ASD_control group (*p* = 0.03184 *), whereas no significant changes were observed in the diet or probiotic groups.

**Table 6:** Topological and functional features of immune–microbial networks before and after anti-inflammatory diet intervention.

Feature	PRE-Intervention	POST-Intervention
Predominant color	Red (negative correlations)	Green (positive correlations)
Central Nodes	*IL-18*, *IP-10*, *IL-8*, *IFN-γ*	*IL-8*, *MIP1-β*, *SDF-1α*, *Eotaxin*
Structural change	More disintegrated, antagonistic network	Denser, cooperative network
Featured bacteria	*Hungatella*, *Lachnoclostridium*, *Klebsiella*	*Blautia*, *Ruminococcus*, *Mediterraneibacter*

## Data Availability

Data is contained within the article.
